# A bibliometric analysis of human strongyloidiasis research (1968 to 2017)

**DOI:** 10.1186/s40794-019-0100-1

**Published:** 2019-12-18

**Authors:** Waleed M. Sweileh

**Affiliations:** Department of Physiology and Pharmacology/Toxicology, Division of Biomedical Sciences College of Medicine and Health Sciences, An-Najah National University, Nablus, Palestine

**Keywords:** Human strongyloidiasis, Bibliometric analysis, Keyword mapping, Geographical distribution, Scopus

## Abstract

**Background:**

Strongyloidiasis is a neglected tropical disease (NTD). It is commonly associated with poverty and poor hygiene. Strongyloidiasis poses an unseen global public health problem. The aim of this study was to assess and analyze peer-reviewed literature on human strongyloidiasis to shed light on the evolution, volume, important topics, and key players in the field of human strongyloidiasis.

**Methods:**

A validated bibliometric method was implemented using Scopus database for the study period from 1968 to 2017. The search strategy was developed based on keywords related to strongyloidiasis. Bibliometric indicators and visualization maps were presented.

**Results:**

In total, 1947 documents were found. Retrieved documents received 32,382 citations, an average of approximately 16.6 per document, and an *h*-index of 76. The most frequently encountered keywords in the retrieved literature focused on hyperinfection, diagnosis, prevalence, and ivermectin. The USA led with 540 (27.7%) documents followed by Brazil (139; 7.1%) and Japan (137; 7.0%). When research output was standardized by income and population size, India ranked first (12.4 documents per GDP/capita) followed by the USA (9.1 documents per GDP/capita). The most active journal involved in publishing articles was the *American Journal of Tropical Medicine and Hygiene* (95; 4.8%). In terms of institutions, the *University of Ryukyus* (Japan) was the most active with 62 (3.2%) publications, followed by the *University of Pennsylvania* with 54 (2.8%) publications.

**Conclusion:**

The volume, growth, and international research collaboration in human strongyloidiasis were inadequate given the long history of the disease, the large number of affected people, and the results obtained for other NTDs. Research in human strongyloidiasis needs to be strengthened and encouraged in endemic regions in Southeast Asia and Latin America. International research networking needs to be established to achieve the goals of Sustainable Development Goals in fighting and eradicating NTDs by 2030.

## Background

Strongyloidiasis is a human parasitic infection and one type of soil-transmitted helminthiasis (STH) [1]. It is mainly caused by *Strongyloides stercoralis* and rarely by *Strongyloides fuelleborni* [2]. Strongyloidiasis is a neglected tropical disease (NTD) that is rarely recognized as a serious public health issue [3–5]. The history of strongyloidiasis goes back to the late nineteenth century, but interest in the condition increased in the 1940s when it was discovered that strongyloidiasis in people with impaired immune function might develop into hyperinfection syndrome and disseminated strongyloidiasis [6]. Much global efforts have been made to fight and eliminate NTDs since more than a billion people, mostly in developing countries, are infected with one or more of the NTDs [7, 8].

Strongyloidiasis is a global disease with a high prevalence in Latin America, Southeast Asia, Sub-Saharan Africa, and low prevalence in certain parts of the Southeast United States [9–11]. A study on the global distribution of *S. stercoralis* indicated that in Africa, the range of infection rates in the communities varies from 0.1% up to 91.8% while in South- and Central-America, the range varies from 1.0 to 75.3% [12]. The same study indicated that in South-East Asia, the infection rate also varies but within a small range. For example, the rate in Cambodia was 17.5%, while that in Thailand and Lao People’s Democratic Republic was 23.7 and 26.2% respectively. At the country level, the highest prevalence of strongyloidiasis was reported from Dominica, Colombia, Argentina, Bangladesh, Ecuador, and Ivory Coast [12]. The low rates reported from certain countries does not mean the absence of the infection. It could be due to lack of screening or diagnostic services particularly in countries with fragile health system and limited resources.

The main predisposing factors for *S. stercoralis* infection are immunosuppressive therapy, human immunodeficiency virus infection (HIV), corticosteroids, HTLV-1-co-infection, malignancies, and organ transplantation [13, 14]. Strongyloidiasis manifests in a wide range of symptoms including dermatological, respiratory (Löffler's syndrome), and digestive complaints. In hyperinfection syndrome and disseminated strongyloidiasis, symptoms may appear in several organs, including the central nervous system [1, 3, 12, 15]. In immunocompetent individuals, most infections with *S. stercoralis* are asymptomatic [16].

Bibliometric analysis, a well-established research method in information science, has been commonly used to shed light on research activity through quantitative description of literature in a particular disease or group of diseases [17–20]. Several bibliometric studies have been published on NTDs [21–23]. However, up to the author’s best knowledge, none was carried out on strongyloidiasis. Therefore, the current study aimed to assess the global research output on human strongyloidiasis published in peer-reviewed journals. The ultimate goals of the current study were to fill the knowledge gap regarding (1) quantitative analysis of national and international publications on strongyloidiasis; (2) and to compare the number of publications obtained with those for other NTDs such as leishmaniasis, Chagas disease, and Buruli ulcers.

## Method

In the current study, bibliometric methodology was implemented using SciVerse Scopus database for the study period from 1968 to 2017. Scopus was used due to the advantages it has over other available databases [24]. For example, Scopus has more than 23,000 indexed journals. This is larger than the number of indexed journals in Web of Science and 100% inclusive of journals in Medline. Furthermore, Scopus has many functions that facilitate bibliometric analysis and that is why most bibliometric studies were carried out using Scopus database [25–29]. The study period was set to represent half a century of research. This study period has witnessed advancement in microbiological and parasitological fields. Furthermore, in the past 50 years migration from various world regions to the modern world was most evident [30].

### Search strategy

The search strategy was based on the use of keywords relevant to *S. stercoralis* (Additional file 1). Examples of keywords used in the search strategy included “S* stercoralis” or “S* f*lleborni” or strongyloid* or “larva currens”. These keywords were used in the title search. Other lesser specific keywords were used in the title search but were followed with certain constraints. Examples of less specific keywords include “Hyperinf* Syndrome” or “soil-transmitted helminthiasis” followed by the presence of the keyword “strongyloid*” in the abstract of the same document. The keyword “disseminated” was not included in the search strategy because other keywords such as strongyloid* and hyperinf* will retrieve the documents about disseminated strongyloidiasis. The quotation marks were used in the search strategy to limit the search to the exact word or phrase while the asterisks were used as a wildcard. An exclusion step was used in the search strategy to eliminate false-positive results. Examples of excluded keywords include seals, dogs, horse, cat, cow, sheep, goat, or camel. This step was implemented to restrict the search to human strongyloidiasis. The current study was not limited to any language. However, only documents published in peer-reviewed journals were analyzed. Therefore, books and book chapters were excluded. The overall search strategy was developed by the author depending on systematic reviews and review articles which included the most common keywords used in the current study. A scheme showing the number of retrieved documents for each step in the search strategy was shown in the supplementary files (Additional file 2).

### Validation of the search strategy

The validation of the search strategy was carried out using the same approach adopted in previously published bibliometric studies [31]. Such approach depended on the absence of false-positive results in the top 200 cited documents. The approach also depended on the absence of false-negative (missing entries) results by comparing the number of documents obtained for certain active authors with those provided by manual search using the author’s name in Scopus database. Agreement in the numbers, tested by interclass correlation coefficient using Statistical Package for Social Sciences (SPSS) [32–36] was used as a validity check for the absence of false-negative results.

### Bibliometric indicators

The analysis included determining the volume and growth of retrieved literature, most active countries, institutions, journals, and authors being involved in publishing the retrieved documents. In Scopus, an article with all authors having the same country affiliation, is counted once for that country. However, if an article has different authors with different country affiliations, then the article is counted once for each country affiliation. Therefore, if we add up the number of publications for each country, the total might exceed the number of retrieved documents because a single article might be counted twice based on author country affiliation on that article. The same applies to counting institutions. Wherever the affiliation of an institution is mentioned in the affiliation of any article, then it was counted once for that institution. Citation analysis was used to show the mean number of citations per document as an index of readership and interest in the retrieved literature. The commonly used Hirsch index (*h*-index) was also used to assess the scientific impact of the retrieved literature [37]. The number of publications produced by each country was standardized by gross domestic product (GDP) per capita obtained from World Bank [38].

### Data visualization

The retrieved data were also analyzed and presented as visualization maps using the free on-line program, VOSviewer [39, 40]. Visualization maps were used to show most frequent keywords in the retrieved documents. The frequency of occurrence of a particular word was directly proportional to the node size presenting the keyword in the map [39, 40]. Visualization maps were also used to shed light on the extent of international collaboration. The VOSviewer calculates the strength of collaboration between any two countries based on the thickness of connecting line and the number of publications. The link strength is given by the program and is not calculated by the author. The higher the link strength, the stronger the collaboration between two countries in terms of the number of co-authored publications relative to other countries.

## Results

In the current study, the interclass Pearson correlation coefficient (r) between number of documents for active author retrieved by the two methods mentioned in the methodology section was 0.96% and the *p*-value was 0.002 indicative of a high degree of validity of search strategy.

### Volume and growth of publications

**Fig. 1:**
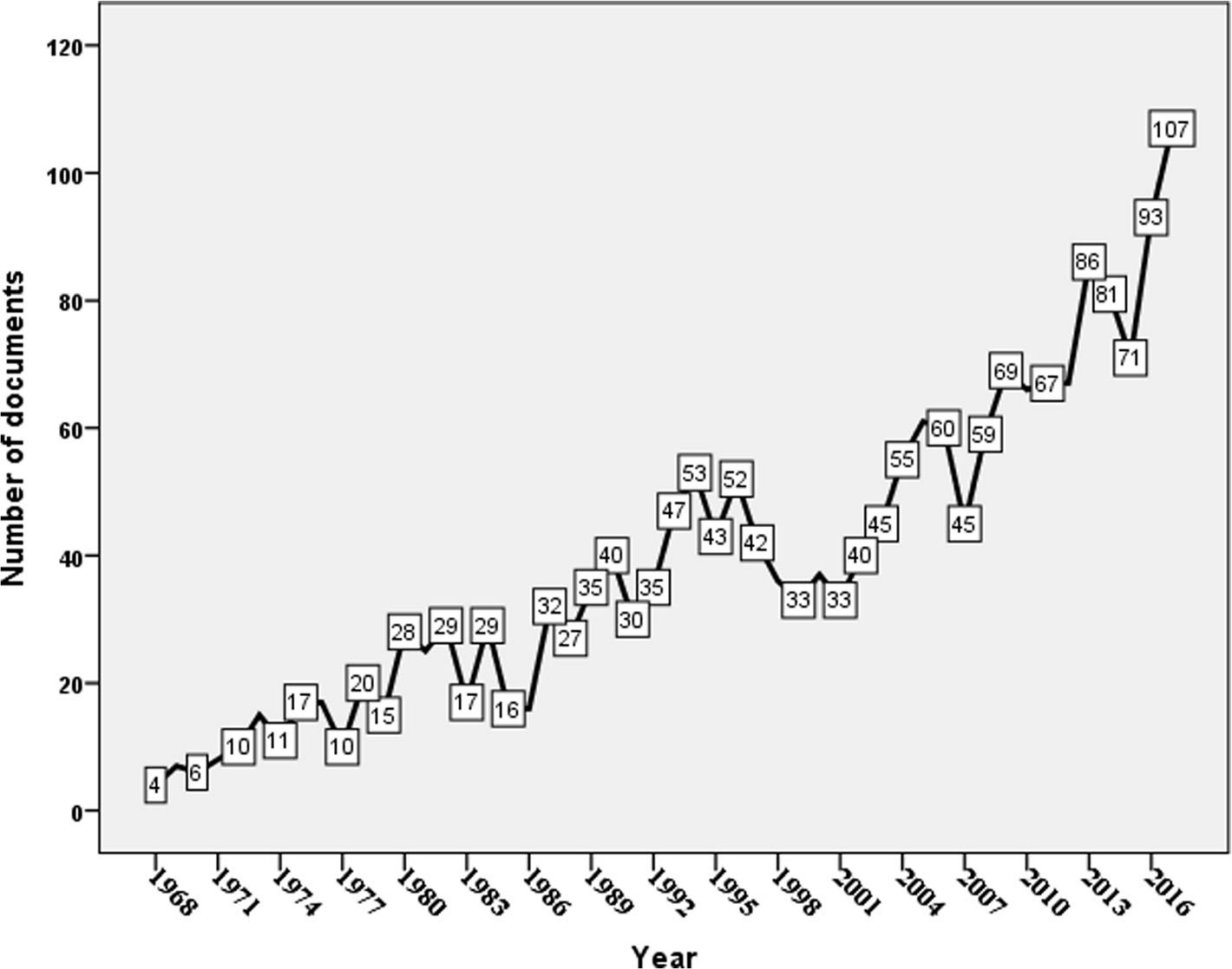
Growth of publications on human strongyloidiasis from 1968 to 2017

In total, 1947 documents were obtained. The first peer-reviewed document appeared in 1908 in *Southern Medical Journal* and was about three cases of *S. stercoralis* in Tennessee, USA [41]. The total number of documents published from 1908 until 1967 was 70 documents (data not shown) and that is why the time of the study was set from 1968. The annual number of publications showed many fluctuations from the early 1970s up to 2017 and never exceeded 100 documents per year except in 2017 (Fig. 1).

### Ten most cited documents

**Fig. 2:**
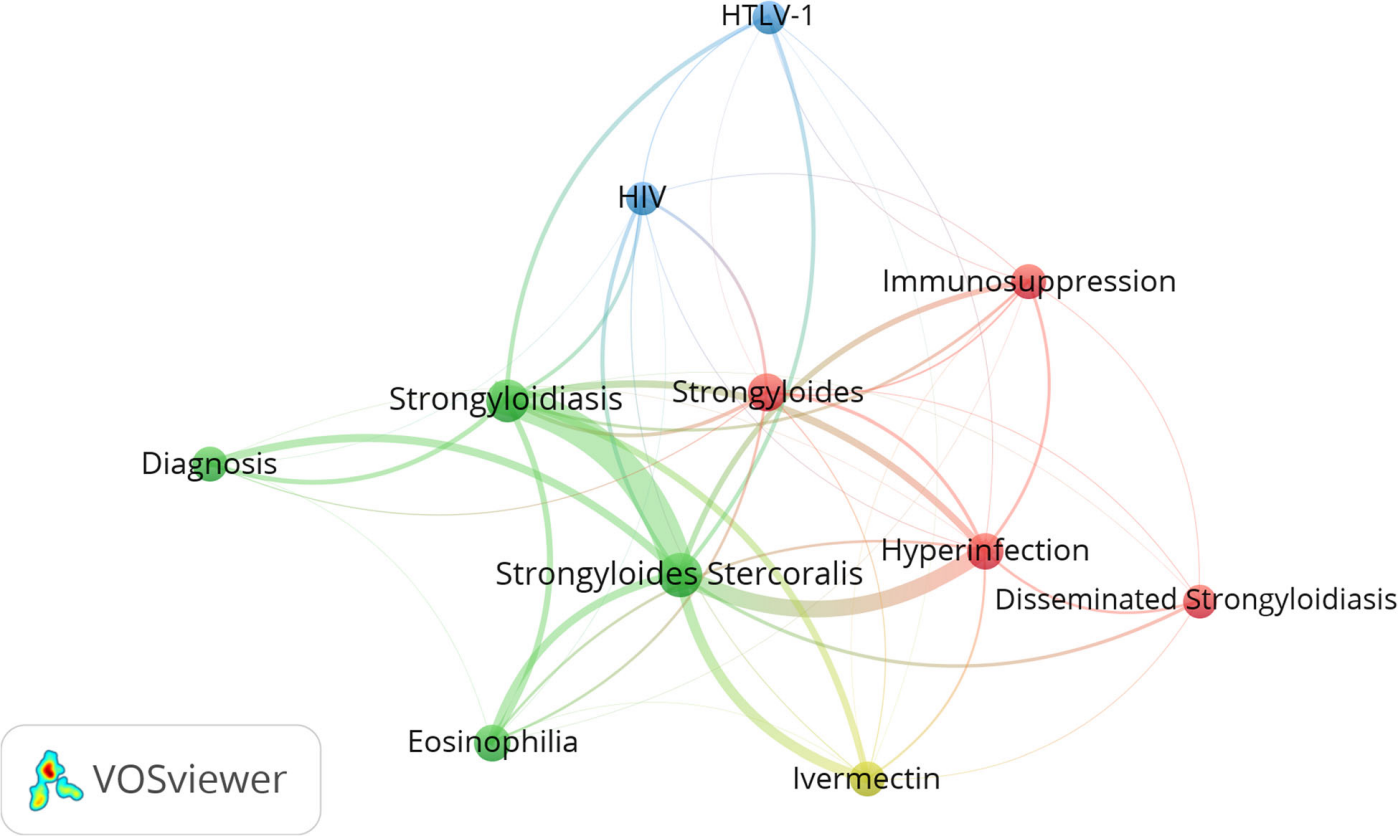
Visualization of most frequent author keywords (minimum occurrences of 20 times)

The retrieved documents received 32,382 citations, an average of approximately 16.6 citations per document. The *h*-index of the retrieved documents was 76. The top ten cited documents [9, 42–50] were shown in Table 1**.** The most cited document was a review article about “*Strongyloides stercoralis* in the Immunocompromised Population“published in *Clinical Microbiology Review* in 2004 [46]. However, when the number of citations was standardized by time, an article published in *PLoS Medicine* received the highest number of citations per year [51] followed by an article published in *PLoS Neglected Tropical Diseases* [12]. The list of highly cited documents included eight review articles and two research articles; one was a clinical trial on the efficacy of ivermectin [42] while the second one was about the multiple stool sampling as a diagnostic approach to detect *S. sterocralis* [52]. The content of the top 10 cited articles was also evident when mapping most frequent author keywords (Fig. 2).

**Table 1:** Ten most cited articles in human strongyloidiasis

Rank	Authors	Title	Year	Number of citations	Number of citations per year^a^	Source title	Reference
1	Keiser, P.B., Nutman, T.B.	Strongyloides stercoralis in the Immunocompromised Population	2004	554	42.6	*Clinical Microbiology Reviews*	[46]
2	Siddiqui, A.A., Berk, S.L.	Diagnosis of Strongyloides stercoralis infection	2001	540	33.8	*Clinical Infectious Diseases*	[44]
3	Olsen, A., van Lieshout, L., Marti, H., Polderman, T., Polman, K., Steinmann, P., Stothard, R., Thybo, S., Verweij, J.J., Magnussen, P.	Strongyloidiasis - the most neglected of the neglected tropical diseases?	2009	278	34.8	*Transactions of the Royal Society of Tropical Medicine and Hygiene*	[48]
4	Genta, R.M.	Global Prevalence Of Strongyloidiasis: Critical Review With Epidemiologic Insights Into The Prevention Of Disseminated Disease	1989	266	9.5	*Reviews of Infectious Diseases*	[9]
5	Igra-Siegman, Y., Kapila, R., Sen, P., Kaminski, Z.C., Louria, D.B.	Syndrome of hyperinfection with Strongyloides stercoralis.	1981	243	9.3	*Reviews of Infectious Diseases*	[49]
6	Scowden, E.B., Schaffner, W., Stone, W.J.	Overwhelming strongyloidiasis: An unappreciated opportunistic infection	1978	231	5.9	*Medicine (United States)*	[45]
7	Strunz, E.C., Addiss, D.G., Stocks, M.E., Ogden, S., Utzinger, J., Freeman, M.C.	Water, Sanitation, Hygiene, and Soil-Transmitted Helminth Infection: A Systematic Review and Meta-Analysis	2014	226	75.3	*PLoS Medicine*	[50]
8	Marti, H., Haji, H.J., Savioli, L., Chwaya, H.M., Mgeni, A.F., Ameir, J.S., Hatz, C.	A comparative trial of a single-dose ivermectin versus three days of albendazole for treatment of Strongyloides stercoralis and other soil- transmitted helminth infections in children	1996	213	10.1	*American Journal of Tropical Medicine and Hygiene*	[42]
9	Knopp, S., Mgeni, A.F., Khamis, I.S., Steinmann, P., Stothard, J.R., Rollinson, D., Marti, H., Utzinger, J.	Diagnosis of soil-transmitted helminths in the era of preventive chemotherapy: Effect of multiple stool sampling and use of different diagnostic techniques	2008	206	22.9	*PLoS Neglected Tropical Diseases*	[43]
10	Schar, F., Trostdorf, U., Giardina, F., Khieu, V., Muth, S., Marti, H., Vounatsou, P., Odermatt, P.	Strongyloides stercoralis: Global Distribution and Risk Factors	2013	200	50.0	*PLoS Neglected Tropical Diseases*	[12]

^a^ Number of citations per year was calculated by dividing the total number of citations received by the number of years from the time of publications up to 2017

### Most active countries

Retrieved data indicated that the USA led with 540 (27.7%) documents. When data was standardized by income and population size, India ranked first followed by Brazil. Top ten active countries were listed in Table 2**.** Regarding citations, documents produced by researchers from Switzerland received the highest number of citations per document (50.2) while those from India received the lowest citations per document (6.3).

**Table 2:** Most active countries in research about human strongyloidiasis

Rank	Country	Frequency *N* = 1947	%	GDP (nominal)/Capita (1000)	^b^Number of publications per GDP/Capita	Number of citations per document
1	United States	540	27.7	59.5	9.1	26.4
2	Brazil	139	7.1	15.8	8.8	14.6
3	Japan	137	7.0	38.4	3.6	18.2
4	United Kingdom	117	6.0	43.9	2.7	28.1
4	France	101	5.2	42.8	2.5	10.4
6	Italy	91	4.7	40.0	2.3	14.9
7	India	87	4.5	7.0	12.4	6.3
8	Spain	86	4.4	38.1	2.3	16.3
9	Australia	74	3.8	47.0	1.6	20.2
10	Switzerland	55	2.8	65.0	0.85	50.2

^b^Gross domestic product (GDP) (nominal) per capita obtained from World Bank data (2017) and expresses in US dollars

### International research collaboration

**Fig. 3:**
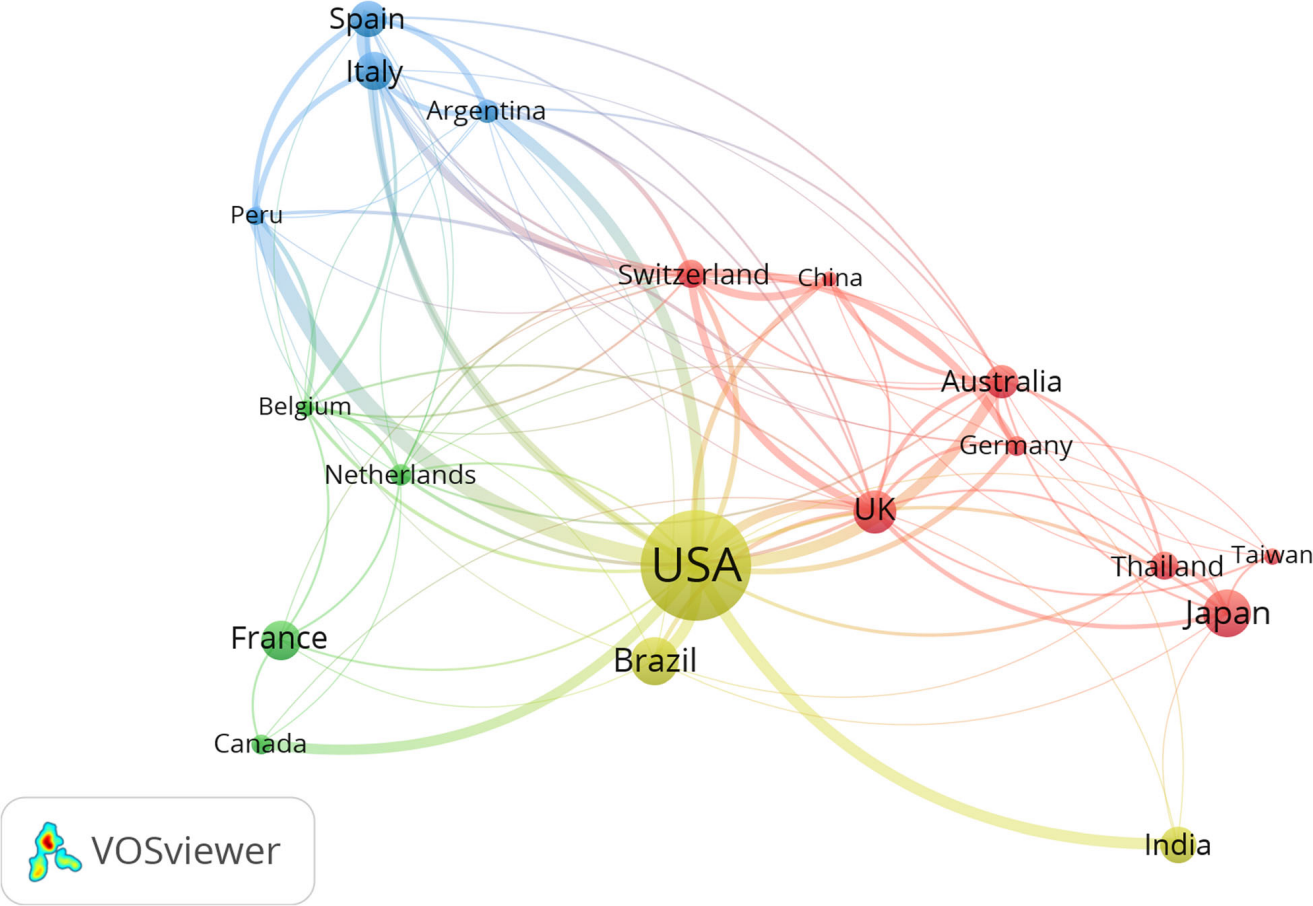
Research collaboration among countries with minimum research output of 20 documents in human strongyloidiasis (1968–2017)

Countries with a minimum research output of 20 documents and had research collaboration with other countries were mapped (Fig. 3**).** The collaboration map showed four clusters of countries. In the collaboration map, all connecting lines were relatively thin indicative of the absence of strong research collaboration among active countries. However, the strongest research collaboration was between the USA and countries in Latin America, particularly Peru (link strength = 14), Brazil (link strength = 10), and Argentina (link strength = 10).

### Ten most active institutions, journals, and authors

The ten most active institutions were shown in Table 3. The *University of Ryukus* (Japan) led with 62 (3.2%) documents. The *University of Pennsylvania* (54; 2.8%) ranked second while the *National Institutes of Health* (Bethesda, USA) ranked third with (46; 2.4%). The list also included active institutions in Switzerland, Brazil, Peru, and Thailand.

**Table 3:** Ten most active institutions in human strongyloidiasis

Rank^a^	Institution	Frequency *N* = 1947	%	Country affiliation
1	*University of the Ryukyus*	62	3.2	Japan
2	*University of Pennsylvania*	54	2.8	USA
3	*National Institutes of Health, Bethesda*	46	2.4	USA
4	*Sacro Cuore Hospital - Don Calabria/ Ospedale Sacro Cuore Don Calabria*	43	2.2	Italy
5	*Universitat Basel and Swiss TPH*	39	2.0	Switzerland
6	*Universidade de Sao Paulo - USP*	31	1.6	Brazil
7	*Liverpool School of Tropical Medicine*	27	1.4	UK
8	*Universidade Federal de Uberlandia*	25	1.3	Brazil
9	*Khon Kaen University*	23	1.2	Thailand
9	*Universidad Peruana Cayetano Heredia*	23	1.2	Peru

^a^Equal institutions were given the same ranking number and then a gap was left in the ranking numbers

The ten most active journals were shown in Table 4. The *American Journal of Tropical Medicine and Hygiene* led with 93 (4.8%) documents followed by *Transactions of The Royal Society of Tropical Medicine and Hygiene* (53; 2.7%) and *Plos Neglected Tropical Diseases* (41; 2.1%). The list of active journals included ones affiliated with the USA, UK, Thailand, the Netherlands, and Poland. The most active journals were mainly in the field of parasitology or tropical medicine.

**Table 4:** Ten most active journals in publishing documents on human strongyloidiasis

Rank^a^	Journal	Frequency *N* = 1947	%	Country affiliation
1	*American Journal Of Tropical Medicine And Hygiene*	93	4.8	USA
2	*Transactions Of The Royal Society Of Tropical Medicine And Hygiene*	53	2.7	UK
3	*Plos Neglected Tropical Diseases*	41	2.1	USA
4	*Journal Of Parasitology*	24	1.2	USA
5	*Acta Tropica*	23	1.2	Netherlands
5	*Southeast Asian Journal Of Tropical Medicine And Public Health*	23	1.2	Thailand
7	*Parasitology*	22	1.1	UK
7	*Southern Medical Journal*	22	1.1	USA
7	*Clinical Infectious Diseases*	22	1.1	USA
10	*Annals Of Tropical Medicine And Parasitology (Pathogens and Global Health)*	20	1.0	UK
10	*International Journal For Parasitology*	20	1.0	Netherlands
10	*Wiadomosci Parazytologiczne (Annals of Parasitology)*	20	1.0	Poland

^a^Equal journals were given equal ranks and a gap was left in the ranking system

Analysis of the retrieved documents showed that 7178 author names were involved in publishing the retrieved documents, giving a mean of 3.6 authors per document taking into consideration that there were 1656 (82.8%) documents as research articles and the remaining 343 (17.2%) documents were as letters, reviews, notes, editorials, conference papers, and short surveys. The list of most active authors (Table 5) included six from the USA, one from Brazil, two from Italy, and one from Japan.

**Table 5:** Ten most active authors in human strongyloidiasis

Rank	Author Name	Frequency *N* = 1947	%	citations	Number of citations per document	Country affiliation as shown in Scopus
1	Lok, J.B.	32	1.6	701	21.9	USA
2	Schad, G.A.	28	1.4	738	26.4	USA
3	Neva, F.A.	27	1.4	1319	48.9	USA
4	Genta, R.M.	26	1.3	1074	41.3	USA
4	Bisoffi, Z.	26	1.3	850	32.7	Italy
6	Costa-Cruz, J.M.	25	1.3	468	18.7	Brazil
7	Nutman, T.B.	23	1.2	1376	59.8	USA
8	Sato, Y.	22	1.1	589	26.8	Japan
9	Buonfrate, D.	21	1.1	750	35.7	Italy
10	Nolan, T.J.	20	1.0	522	26.1	USA

### Research themes of the retrieved documents

**Fig. 4:**
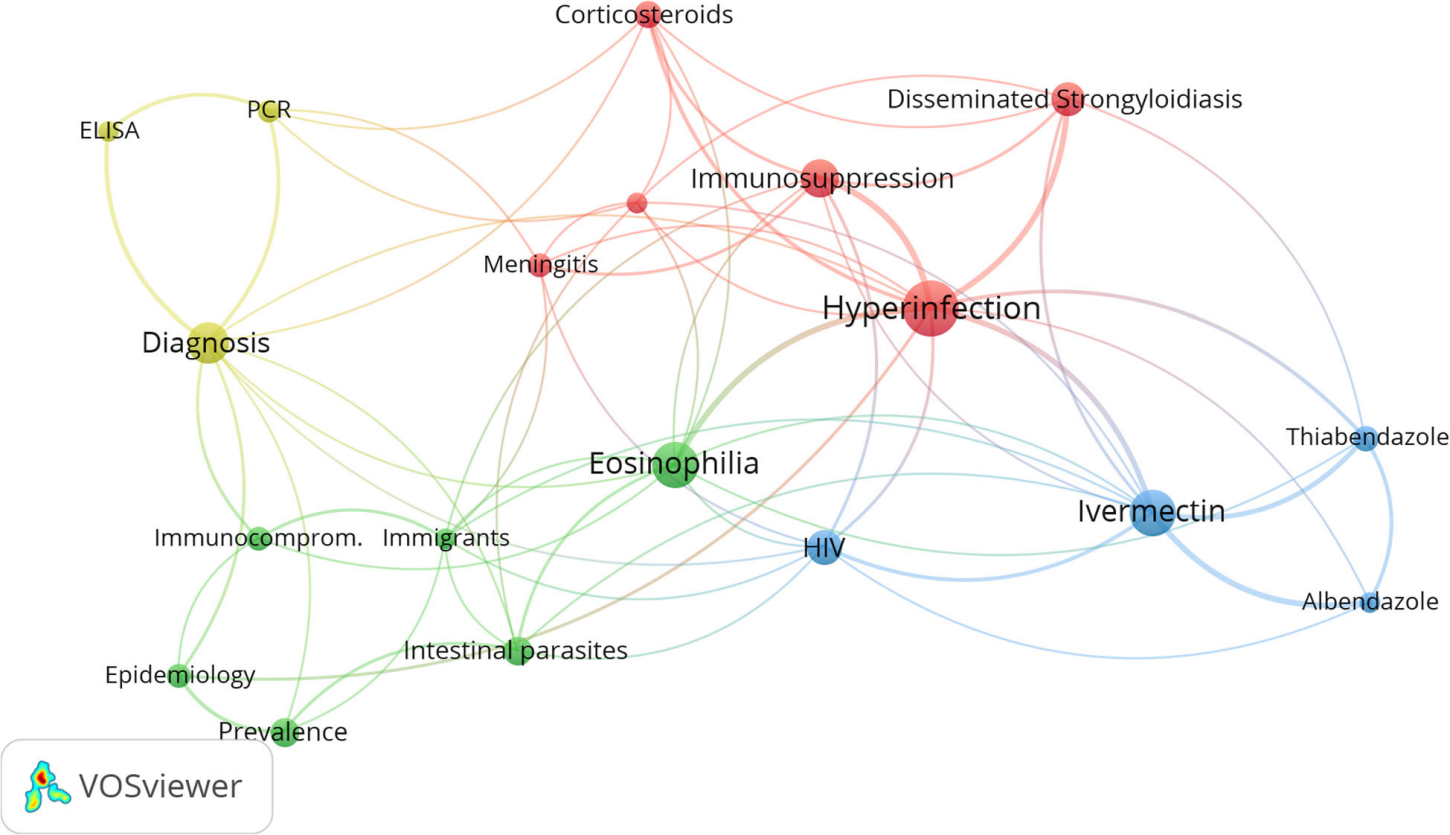
Visualization map of most frequent terms in titles/abstracts of the retrieved documents

The main research themes in the retrieved documents were investigated by visualizing terms used by authors in titles and abstracts with a minimum occurrence of 20 times. The map showed that the most frequent terms in titles/abstracts created four clusters (Fig. 4). The first cluster (red) focused on immunosuppression and corticosteroids (512 documents) as risk factors for hyperinfection and disseminated strongyloidiasis. The second cluster (green) was mainly about epidemiology/prevalence (241 documents) of the disease. The third cluster (blue) focused mainly on treatment using ivermectin and other medications (546 documents). The fourth cluster (light green) focused on diagnosis and new techniques such as PCR and ELISA (347 documents).

## Discussion

The current study aimed to assess global research output on human strongyloidiasis hoping to (1) attract the attention of researchers and health policy makers to this NTD, (2) add information to the existing literature on strongyloidiasis, and (3) give credit to key players in strongyloidiasis research.

### Volume of the retrieved literature

The current study indicated that the volume of the retrieved literature was relatively low given that 30–100 million individuals or more might be infected with *S. stercoralis* and given the number of publications on other neglected tropical diseases [1, 53, 54]. For example, a bibliometric study on leishmaniasis using Scopus for the study period from 1998 to 2017 yielded 17,570 publications [55]. Another study on dengue found that the number of publications reached 1000 publications in one year [56]. A third study showed that contribution of countries in Latin America to Chagas diseases was approximately 3000 articles [57]. There are several reasons behind the relatively low volume of literature on strongyloidiasis compared to other NTDs. The number of experts and those specifically interested in *S. stercoralis* maybe limited. Even if the number of experts and researchers in this field were high, the limited international research collaboration might have played a negative role in global research output [58, 59]. Research networking and collaboration is an essential aspect to make scientific progress, particularly in the biomedical field. This was evident in the co-authorship as well as the multidisciplinary nature of recent science [60, 61]. Establishing research networks increases research output, creates new research opportunities, facilitates technology transfer, increase citations of research manuscripts, especially if there is an international team of authors involved [62–65]. Building research networks is extremely important for developing countries where research community is fragmented and research grants are scarce [62]. The finding that the *h*-index of the retrieved literature on strongyloidiasis was 76 which is relatively lower than that reported for dengue [66] and leishmaniasis [55].

The wrong belief that strongyloidiasis does not pose a global health threat might also have played a negative role in this regard [67]. Research interest in emerging serious infections such as Zika and Ebola and other endemic infections such as malaria and tuberculosis might have overshadowed research in infections such as soil-transmitted helminthiasis (STH) [68].

### Annual growth of publications

The current study also showed that more than half of the retrieved documents were published in the past two decades. Opportunistic infections in certain categories of people such as patients with hematological malignancies, transplant recipients, and patients on corticosteroids or other immunosuppressive drugs [69–72] positively affected the growth of publications in this field in the past two decades. The advancement in techniques implemented in the diagnosis and detection of *S. stercoralis* was also an indirect potential reason behind the increasing number of publications seen in the past two decades [73–75]. Several international programs such as the World Health Organization’s Global NTD Programs, the Centers for Disease Control and Prevention’s Global NTD Program, the United States Global Health Initiative, the United States Agency for International Development’s NTD Program, and others were implemented to focus on NTDs, and fighting to control or eliminate them [76–78]. It is hoped that these programs will stimulate researchers and increase the volume and growth of publications in strongyloidiasis and other NTDs. The starting of several peer-reviewed journals in the field of neglected diseases as well as diseases of poverty helped in the growth and visibility of strongyloidiasis research in recent years. Examples of such journals include *PLOS Neglected Tropical Diseases* and *Infectious Diseases of Poverty*. Furthermore, the emergence of ivermectin as an effective drug of choice for acute and chronic strongyloidiasis in intestinal stages, hyperinfection syndrome, and disseminated strongyloidiasis positively affected the number of publications in the past two decades [42].

The movement of large numbers of migrants from low- to high-income countries [79] and the presence of large numbers of patients with an immune problem who are at high risk of strongyloidiasis changed the attitude of developed countries toward this infection and positively affected the growth of publications in this field [15, 80].

### Highly cited documents

The current study showed that highly cited documents in strongyloidiasis focused on hyperinfection syndrome, prevalence, diagnosis, and treatment. The fatal consequences of disseminated strongyloidiasis and hyperinfection syndrome were the main reason behind the increasing emphasis on strongyloidiasis literature. Detailed data on the epidemiology of *S. stercoralis* are also needed and constitute a real challenge to international health bodies [1]. Such epidemiological data are needed not only in endemic areas but also in developed countries in Europe and Northern America. It is strongly believed that human strongyloidiasis is underdiagnosed because many cases are asymptomatic and the available diagnostic methods lack sensitivity [1]. For example, in many developed countries, immigrants and refugees particularly those migrating from tropical and subtropical countries need to be screened for strongyloidiasis [81–83]. Furthermore, the large numbers of people with HIV/AIDS, HLTV-1, and people on immunosuppressants due to organ transplant requires more accurate and detailed screening [84–91]. The number of positive cases, and consequently the number of publications, could be partly due to implementing serological and molecular methods in immune-compromised patients or high-risk groups [75].

### Most active countries

The current study indicated that the USA led in terms of number of publications, number of active institutions, journals, and authors. Several reasons could be cited for this leadership. First, the relatively high number of researchers, academic and research institutions, technology and funding. Second, strongyloidiasis have been detected in the USA in certain rural areas of the southeastern states and the Appalachian region [17]. Third, the large numbers of Asian and Latino migrants and refugees in the USA [79] increased the interest of USA researchers in this infection. Fourth, the presence of immunocompromised people in the USA and the high risk of this category to develop hyperinfection syndrome increased the interest of USA researchers in this disease. The list of active countries also included several European countries and Australia. The argument made about the role of USA in research in human strongyloidiasis applies to active European countries and Australia. The list of active countries included one country in Latin America, Brazil. A systematic review of the prevalence of strongyloidiasis in Latin America reported that high rates of strongyloidiasis are present in Argentina, Ecuador, Venezuela, Peru and Brazil [92]. The authors of the systematic review concluded that for most studied countries it was not possible to define reliable prevalence data because of paucity and/or inadequacy of studies and the need for specific diagnostic methods for detection of *S. stercoralis* [92]. The list of active countries included Japan and India. The most active institution was also based in Japan. A recent study that reviewed studies of the last 20 years on *S. stercoralis’s* global prevalence found that in South-East Asia and the Western Pacific region, 40 investigations were conducted in Thailand, 15 in Australia, 14 in Japan, and 14 in India [12]. In Japan, studies have shown that *S. stercoralis* was only endemic in Okinawa prefecture mostly due to high prevalence of HTLV-1 infections [93, 94].

## Limitations

The current study has a few limitations that are inherent to bibliometric methodology. Scopus is not inclusive of all parasitology and infectious-related journals, particularly those published from developing countries in Southeast Asia, Latin America, Eastern Europe, and Africa. Most countries with a high prevalence of the infection have lesser number of indexed journals than high-income countries where prevalence is supposed to be low or absent. Furthermore, the number of non-English journals indexed in Scopus is relatively low which creates a bias toward countries publishing English journals. The search strategy implemented in the current study was meant to be comprehensive and valid. However, the presence of false-positive or false-negative documents remains a possibility because search strategy showed more than 95% validity (agreement) and not 100% agreement between results for active authors and results obtained by searching authors individually. Finally, the method of counting documents by Scopus allows the same document to be counted several times if authors have different country affiliations. The same applies when counting the number of documents for authors and institutions. This means that there is an overlap in the results pertaining to top ten active countries, authors and institutions. Therefore, the results might have overestimated the real research productivity of certain countries, authors, or institutions.

## Conclusion

The current study showed that the volume and growth of literature in strongyloidiasis were relatively poor given the large number of affected people worldwide. The current study also showed limited international research collaboration in this field. Certain world regions in Africa, the Middle East, and Eastern Europe showed a negligible contribution to this field. To support and strengthen the fight against NTDs including strongyloidiasis and to implement the paradigm of universal health coverage (SDG target 3.8), international bodies need to stimulate researchers to conduct research activities in all aspects of NTDs [54]. The current study supplies the World Health Organization and other national and international health bodies with data needed to plan future activities that will help eliminate this neglected infection. Furthermore, the findings of the current study help create a forum that brings together all those interested in the subject to unify efforts and recruit funding needed to win the fight against strongyloidiasis.

## Supplementary information

**Additional file 1.** Search strategy and keywords used

**Additional file 2.** A scheme showing search strategy with the number of documents retrieved in each step.

## Data Availability

Data pertaining to this study could be retrieved using Scopus and the search strategy is outlined in Additional file 1.

## Abbreviations

NTDs: Neglected tropical diseases
STH: Soil-transmitted helminthiasis
