# Supplementary material for: A bibliometric analysis of human strongyloidiasis research (1968 to 2017)
Source: Trop Dis Travel Med Vaccines. 2019 Dec 18;5:24. doi: 10.1186/s40794-019-0100-1 (PMC6921599; doi:10.1186/s40794-019-0100-1)
Supplement: Supplementary file 1 — Additional file 1. Search strategy and keywords used [file 40794_2019_100_MOESM1_ESM.docx]

**A bibliometric analysis of human strongyloidiasis research (1968 to 2017)**

**Waleed M. Sweileh**

Department of Physiology, Pharmacology/Toxicology,

Division of Biomedical Sciences,

College of Medicine and Health Sciences,

An-Najah National University,

Nablus, Palestine

E-mail: [waleedsweileh@yahoo.com](mailto:waleedsweileh@yahoo.com)

Tel: + 970-599-225906

**Additional file 1: Search strategy and keywords used in Scopus.**

1. TITLE ( "intestinal parasit*"  OR  nematod*  OR  "round worm"  OR  "geo-helminth*"  OR  geohelminth*  OR  "soil-transmitted *helminth*"  OR  "hyperinfe* syndrome" or “Loeffler's syndrome” or “[Parasitic pneumonia](https://en.wikipedia.org/wiki/Parasitic_pneumonia)” or “Cochin-China diarrhea”)  AND  ABS ( "S* stercoralis"  OR  "S* f*lleborni" )  AND  ALL ( "strongyloidiasis" )
2. TITLE ( strongyloid*  OR  "larva currens"  OR  anguillulose)  AND  ALL ( stercoralis  OR  f*lleborni )
3. NOT  TITLE ( seals  OR  dog*  OR  horse  OR  cat  OR  cow  OR  sheep  OR  goat  OR  camel  OR  pet  OR  animal  OR  bird  OR  vete*  OR  cattle  OR  primateOR  equine OR  sea  OR  foal  OR  ruminant*  OR  ratti  OR  ransomi  or evolut* OR  papillosus  OR  canine  OR  monkey*  OR  cati  OR  venezuelensis  OR  avium  OR  mouse  OR  mice  OR  animal  OR  milk )
4. NOT  SRCTITLE ( veterina*  OR  ruminant  OR  animal  OR  kleintierpraxis  OR  zoology  OR  primat*  OR  wildlife  OR  livestock  OR  zoo*  OR  evolution  OR  agricultur*  OR  invertebrate )
5. LIMIT-TO ( SRCTYPE ,  "j " )
6. EXCLUDE ( DOCTYPE ,  "er " )
7. EXCLUDE ( PUBYEAR ,  2018 )

**Search strategy= #1 OR #2 (AND) #3 (AND) #4 (AND) #5 (AND) #6 (AND) #7**
