# Supplementary material for: A bibliometric analysis of human strongyloidiasis research (1968 to 2017)
Source: Trop Dis Travel Med Vaccines. 2019 Dec 18;5:24. doi: 10.1186/s40794-019-0100-1 (PMC6921599; doi:10.1186/s40794-019-0100-1)
Supplement: Supplementary file 2 — Additional file 2. A scheme showing search strategy with the number of documents retrieved in each step. [file 40794_2019_100_MOESM2_ESM.docx]

**A bibliometric analysis of human strongyloidiasis research (1968 to 2017)**

**Additional file 2** Scheme showing step-by-step Search strategy

TITLE(strongyloid* or "larva currens" or anguillulose) AND ALL(stercoralis or f*lleborni )

**N = 2470**

TITLE("intestinal parasit*" or nematod* or "round worm" or "geo-helminth*" or geohelminth* or "soil-transmitted *helminth*") AND ABS("S* stercoralis" OR "S* f*lleborni") AND ALL("strongyloidiasis")

**N = 153**

**Combine**

**N = 2572**

**EXCLUDE**

AND NOT TITLE(seals or dog* or horse or cat or cow or sheep or goat or camel or pet or animal or bird or vete* or cattle or primate* or equine or sea or foal or ruminant* or ratti or ransomi or papillosus or canine or monkey* or cati or venezuelensis or avium or mouse or mice or animal or milk)) ) AND NOT SRCTITLE(veterina* or Ruminant or animal or Kleintierpraxis or Zoology or Primat* or wildlife or Livestock or zoo* or evolution or agricultur* or Invertebrate ) and not TITLE(evolution) )

**N = 2258**

Limit sources to “Journal documents”

**N = 2234**

Limit study period from 1968 - 2017

**N = 1947**
